# Taxonomic review of the genus *Paradiestrammena* (Orthoptera, Rhaphidophoridae), with descriptions of three new species from Borneo, Malaysia

**DOI:** 10.3897/zookeys.1296.213690

**Published:** 2026-10-29

**Authors:** Qidi Zhu, Amira Aqilah Muhammad, Tony Robillard, Dayang Fazrinah Binti Awg Damit, Razy Japir, Arthur Y. C. Chung, Fuming Shi, Ming Kai Tan

**Affiliations:** 1 College of Agronomy, Jiangxi Agricultural University, Nanchang 330045, China College of Agronomy, Jiangxi Agricultural University Nanchang China; 2 Institute of Biological Sciences, Faculty of Science, Universiti Malaya, Kuala Lumpur 50603, Malaysia Institute of Biological Sciences, Faculty of Science, Universiti Malaya Kuala Lumpur Malaysia; 3 Institut de Systématique, Evolution, Biodiversité (ISYEB), Muséum national d’Histoire naturelle, CNRS, SU, EPHE-PSL, UA, 57 rue Cuvier, CP 50, 75231 Paris Cedex 05, France Institut de Systématique, Evolution, Biodiversité (ISYEB), Muséum national d’Histoire naturelle, CNRS, SU, EPHE-PSL, UA Paris France; 4 Forest Research Centre (Sepilok), Sabah Forestry Department, PO Box 1407, 90715 Sandakan, Sabah, Malaysia Forest Research Centre (Sepilok), Sabah Forestry Department Sandakan Malaysia; 5 College of Life Sciences, Hebei University, Baoding 071002, China College of Life Sciences, Hebei University Baoding China; 6 Lee Kong Chian Natural History Museum, Faculty of Science, National University of Singapore, 2 Conservatory Drive, Singapore 117377, Singapore Lee Kong Chian Natural History Museum, Faculty of Science, National University of Singapore Singapore Singapore

**Keywords:** Aemodogryllinae, Sabah, Sarawak, taxonomy

## Abstract

The genus *Paradiestrammena* Chopard, 1919 (Orthoptera, Rhaphidophoridae, Aemodogryllinae) is mainly distributed in Vietnam, Cambodia, Malaysia, Thailand, Laos, and southern China, with 13 species recorded. Herein, three new species of *Paradiestrammena* are described from Sarawak and Sabah, namely *P. cordata* Zhu & Tan, **sp. nov**., *P. aquilina* Zhu & Tan, **sp. nov**., and *P. sabahensis* Zhu & Tan, **sp. nov**. A key to male *Paradiestrammena* species is provided.

## Introduction

Chopard (1919a) erected the genus *Paradiestrammena* Chopard, 1919 and transferred *Diestrammena feai* Chopard, 1915, *D. brevifrons* Chopard, 1916, *D. annandalei* Kirby, 1908, and *D. gravelyi* Chopard, 1916 to it based on the presence of relatively few, nearly regular spines on the hind tibiae. However, no type species was designated at the time. Subsequently, Chopard (1919b) described an additional species, *P. vitalisi* Chopard, 1919, from Laos.

Karny (1920, 1925) and Chopard (1921) later synonymized *Paradiestrammena* with *Diestrammena* Brunner von Wattenwyl, 1888. Storozhenko (1990) established the subgenus Atachycines (Eutachycines) and designated *D. feai* as its type species. Moreover, *D. brevifrons*, *D. annandalei*, *D. gravelyi*, and *P. vitalisi* were all transferred based on the structure of the male genitalia and the spines of the fore and mid tibiae.

Since the type species of *Atachycines*, Diestrammena (Atachycines) horazumi Furukawa, 1933), was based on a nymph, the classification of *Atachycines* species has been uncertain (Storozhenko 1990; Gorochov and Storozhenko 1992; Gorochov 1994). Consequently, Gorochov (1998) reestablished the genus *Paradiestrammena* and designated *D. gravelyi* as its type species. Moreover, *D. apicalis* Brunner von Wattenwyl, 1888, *P. vitalisi*, *D. sarawakana* Chopard, 1940, and *Atachycines autumnalis* Gorochov, 1994 were transferred to *Paradiestrammena*, and three new species were described at the time: *P. vernalis* Gorochov, 1998 from Vietnam, *P. kotljarovskyi* Gorochov, 1998 from Japan, and *P. tarbinskyi* Gorochov, 1998 from Malaysia. In the same paper, he described two species from Vietnam in nominative subgenus of the genus *Diestrammena*, namely D. (D.) storozhenkoi Gorochov, 1998 and D. (D.) mistshenkoi Gorochov, 1998.

Gorochov (2002a) later described two new species, *Paradiestrammena contumi* Gorochov, 2002 and *P. enata* Gorochov, 2002, and one new subspecies, *P. storozhenkoi khmerica* Gorochov, 2002 from Vietnam, Cambodia, and Thailand, respectively. He also transferred *Diestrammena maculata* Chopard, 1915, *D. storozhenkoi*, and *D. mistshenkoi* to *Paradiestrammena* based on the structure of the female subgenital plate. In the same year, Gorochov (2002b) described two additional species of *Paradiestrammena* from Japan, *P. mira* Gorochov, 2002 and *P. propinqua* Gorochov, 2002.

Sugimoto and Ichikawa (2003) transferred *Diestrammena apicalis* to *Atachycines*, and synonymized *Paradiestrammena kotljarovskyi* with that species after examining the images of the holotype. Ichikawa (2006) subsequently transferred *P. mira* to the genus *Neotachycines* and treated *P. propinqua* as its junior synonym.

Zhu et al. (2020) described two additional *Paradiestrammena* species from China, *P. trigona* Zhu, Lu & Shi, 2020 and *P. xima* Zhu, Lu & Shi, 2020, and provided DNA barcode sequences for each. To date, *Paradiestrammena* comprises 13 valid species distributed mainly in Vietnam, Cambodia, Malaysia, Thailand, Laos, and China (Cigliano et al. 2026).

Based on natural history museum specimens and new material collected from recent field sampling, we describe three new species of *Paradiestrammena*, namely *P. cordata* Zhu & Tan, sp. nov., *P. aquilina* Zhu & Tan, sp. nov., and *P. sabahensis* Zhu & Tan, sp. nov. from Sarawak and Sabah. A key to male *Paradiestrammena* species is provided.

## Materials and methods

Material examined here comprises specimens from the historical collection in the Zoological Reference Collection (**ZRC**) of the Lee Kong Chian Natural History Museum, Singapore, and new material collected in Sabah (East Malaysia) between 2022 and 2026. Whenever possible, live specimens were photographed using a Canon EOS 500D digital SLR camera equipped with an EF 100 mm f/2.8 Macro USM lens, and Canon Macro Twin Lite MT-24EX was used for lighting and flash.

The specimens were preserved in absolute analytical-grade ethanol, and later pinned and dry-preserved. The male genitalia were dissected with an insect needle. Pinned specimens were imaged using a Canon EOS 6D digital SLR camera attached to a Visionary Digital Passport system. Images of morphological features were taken using an MP-E 65 mm f/2.8 USM macro photo lens (1–5×). Image stacking was done using Zerene Stacker v. 1.04. The male genitalia were imaged using a Leica M205C Encoded stereomicroscope. Image stacking was done using Leica Focus Stacking Imaging System operated within Leica Application Suite X. Measurements followed Zhu et al. (2020).

The specimens collected were deposited in:

**FRC** Forest Research Centre (Sepilok), Sabah Forestry Department, East Malaysia

**ZRC** Zoological Reference Collection, Lee Kong Chian Natural History Museum, Singapore

## Results

### Family Rhaphidophoridae Walker, 1869

**Subfamily Aemodogryllinae Jacobson, 1905**

**Tribe Aemodogryllini Jacobson, 1905**

#### Genus Paradiestrammena

Taxon classificationAnimaliaOrthopteraRhaphidophoridae

Chopard, 1919

E2AF0782-EBD8-56B2-B685-1CF8A3E2815D

##### Type species.

*Diestrammena gravelyi* Chopard, 1916, subsequent designation by Gorochov (1998).

*Paradiestrammena* Chopard, 1919a: 375—Karny 1920: 144; Chopard 1921: 516; Karny 1925: 5; Gorochov 1998: 51; Zhu et al. 2020: 1273.

##### Included species.

*Paradiestrammena autumnalis* (Gorochov, 1994) from Vietnam

*Paradiestrammena contumi* Gorochov, 2002 from Vietnam

*Paradiestrammena enata* Gorochov, 2002 from Cambodia

*Paradiestrammena gravelyi* (Chopard, 1916) from Malaysia

*Paradiestrammena maculata* (Chopard, 1916) from Cambodia

*Paradiestrammena mistshenkoi* (Gorochov, 1998) from Vietnam

*Paradiestrammena sarawakana* (Chopard, 1940) from Malaysia

*Paradiestrammena storozhenkoi* (Gorochov, 1998), including *P. s. khmerica* Gorochov, 2002 from Thailand and *P. s. storozhenkoi* (Gorochov, 1998) from Vietnam

*Paradiestrammena tarbinskyi* Gorochov, 1998 from Malaysia

*Paradiestrammena trigona* Zhu, Lu & Shi, 2020 from China

*Paradiestrammena vernalis* Gorochov, 1998 from Vietnam

*Paradiestrammena vitalisi* Chopard, 1919 from Laos

*Paradiestrammena xima* Zhu, Lu & Shi, 2020 from China

##### Distribution.

Cambodia, China, Laos, Malaysia, Thailand and Vietnam.

#### Paradiestrammena cordata

Taxon classificationAnimaliaOrthopteraRhaphidophoridae

Zhu & Tan sp. nov.

8D52DC26-254B-5F18-A106-9270D6686A22

https://zoobank.org/A33F33E2-B83D-4994-B5D0-2F9BDC6697DE

Figs 1, 2A–C

##### Material examined.

***Holotype***: East Malaysia • ♂, Sarawak State, Bau [near Kuching], near Gua. Kopo; 19 February 1997; HH Tan leg.; THH9724 (ZRC).

##### Diagnosis.

The species is similar to *P. tarbinskyi* from Malay Peninsula, from which it can be distinguished by the following characters of male genitalia: apical area of dorsomedian lobe with a concavity (Fig. 2A), while rounded in *P. tarbinskyi*; apical area of sclerite with a slightly shorter, hooked process (Fig. 2A, C), while rather long, thin, and spine-shaped in *P. tarbinskyi*. The new species also differs from its Bornean congener, *P. sarawakana* from Sarawak in its unicolorous body, instead of having the black and brown patterns of *P. sarawakana*.

**Figure 1.**
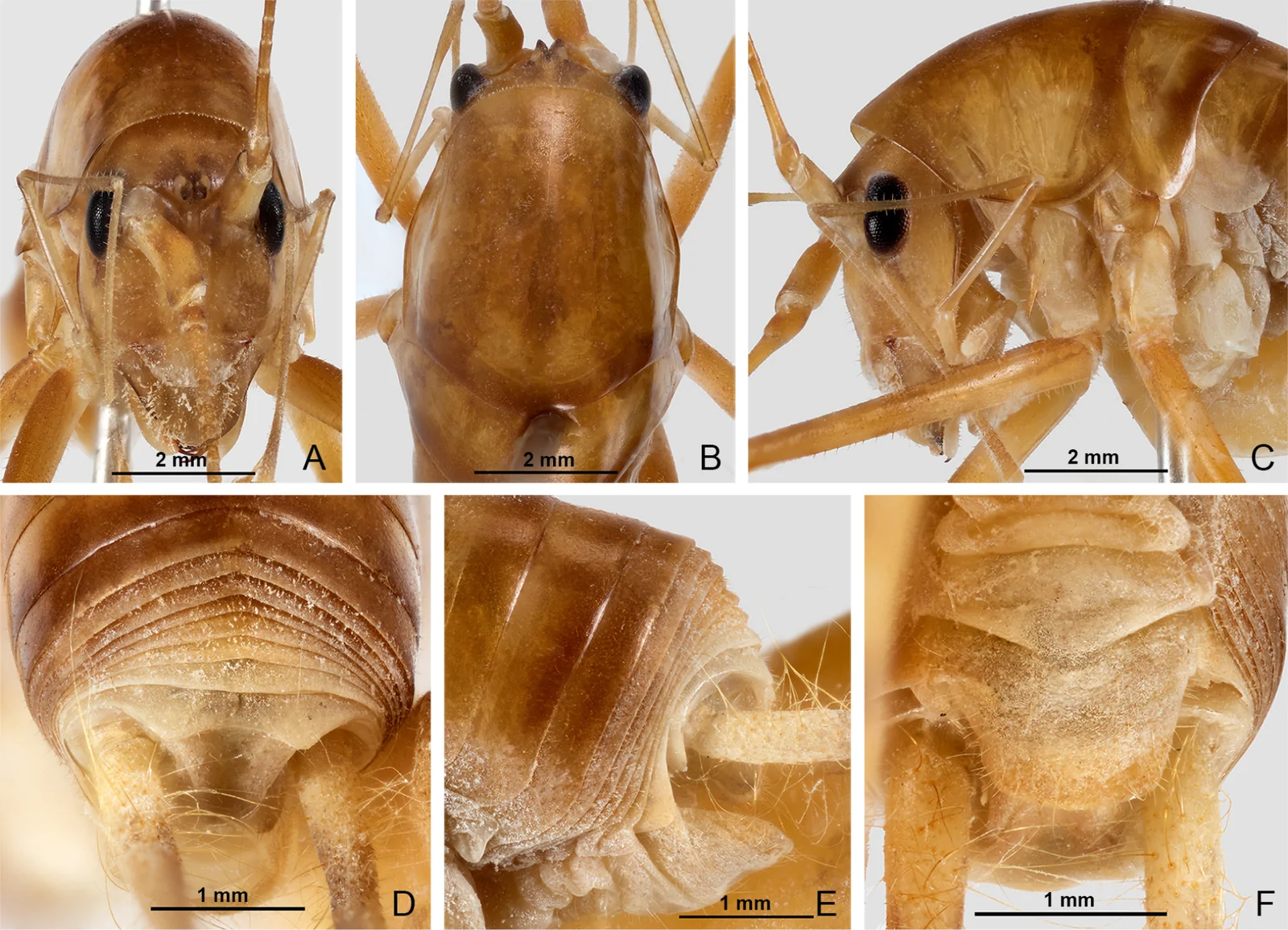
*Paradiestrammena cordata* Zhu & Tan, sp. nov., ♂ holotype (ZRC). **A–C**. Head and pronotum: **A**. Frontal view; **B**. Dorsal view; **C**. Lateral view. **D–F**. Apex of abdomen: **D**. Dorsal view; **E**. Lateral view; **F**. Ventral view.

**Figure 2.**
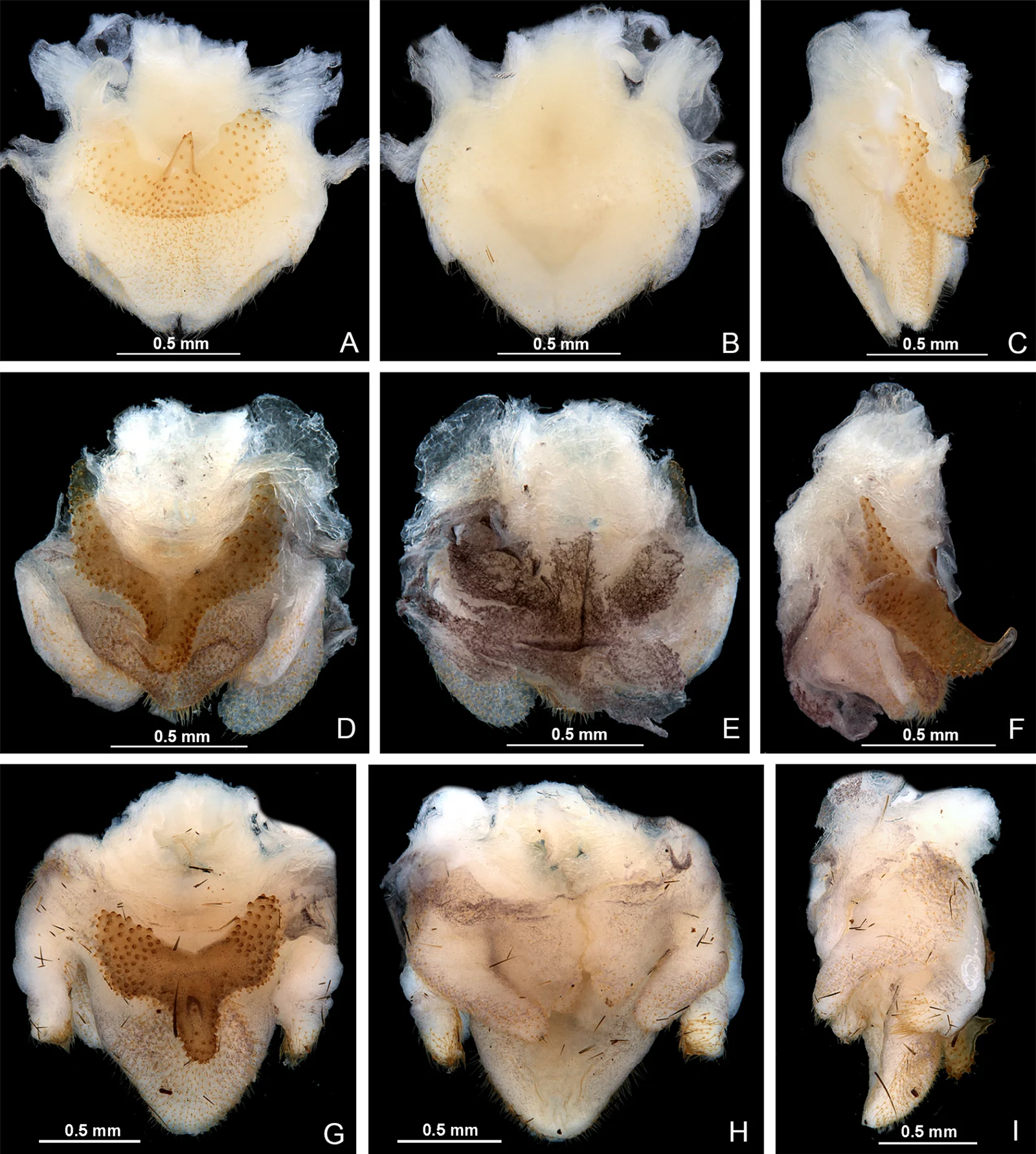
Male genitalia of *Paradiestrammena* spp. **A–C**. *P. cordata* Zhu & Tan, sp. nov.; **D–F**. *P. aquilina* Zhu & Tan, sp. nov.; **G–I**. *P. sabahensis* Zhu & Tan, sp. nov. **A, D, G**. Dorsal views; **B, E, H**. Ventral views; **C, F, I**. Lateral views.

##### Description.

**Male**. Body medium-sized. Fastigium verticis with two conical tubercles; apices separated, pointing forwards. Eyes ovoid, protruding forwards. Lateral ocelli nearly circular, situated on lateral surface of basal rostral tubercles. Apical segment of maxillary palp obviously longer than subapical one; apex globular.

Pronotum long; anterior margin of disc straight; posterior margin arcuate; lateral lobe longer than high; ventral margin arched. Mesonotum and metanotum short; posterior margin of mesonotum arcuate; posterior margin of metanotum straight. Fore coxa with one small spine. Fore femur unarmed on ventral surface; internal genicular lobe with one small spine; external genicular lobe with one long spine. Fore tibia with two inner spines and two outer spines on ventral surface, apex with one outer spine on dorsal surface and a pair of spines on ventral surface with one small spine in between. Middle femur unarmed on ventral surface; internal and external genicular lobes with one long spine each. Middle tibia with one inner spine and two outer spines on ventral surface, apex with one dorsal spine and a pair of ventral spines with one small spine in between. Hind femur with one inner spine on ventral surface; internal genicular lobe with one small spine; external genicular lobe unarmed. Hind tibia with 16 inner and outer spines on dorsal surface each; subapex with a pair of dorsal spines; apex with a pair of dorsal spines and two pairs of ventral spines; interodorsal spine slightly shorter than hind basitarsus. Hind basitarsus with one dorsal spine, with bristles on ventral surface.

Posterior margins of all abdominal tergites straight. Epiproct tongue-shaped. Paraproct triangular in lateral view. Cercus long, conical, apex acute. Genitalia with sclerite, nearly heart-shaped, basal area broadly concave, lateral margin nearly straight, apical area with hooked process, curved upwards, gradually narrowing to apex, apex slightly acute, pointing to upwards and forwards; with six membranous lobes, dorsomedian lobe broad, apical area slightly concave, dorsolateral lobe short and thin, cylindrical, obviously shorter than dorsomedian lobe, ventromedian lobe nearly tongue-shaped, obviously shorter than dorsomedian lobe, apex rounded, ventrolateral lobe cylindrical, nearly equal in length to dorsomedian lobe. Subgenital plate quadrangular, length greater than height, posterior margin rounded.

**Female**. Unknown.

##### Coloration.

Body yellowish-brown. Eyes black. Ocelli yellow. Basal half of hind femur with penniform brown stripes.

##### Measurements.

Body length: ♂ 8.65 mm; length of pronotum: ♂ 4.60 mm; length of fore femur: ♂ 8.70 mm; length of hind femur: ♂ 15.50 mm; length of hind tibia: ♂ 17.65 mm; length of hind basitarsus: ♂ 3.80 mm.

##### Etymology.

The species name is derived from the Latin *cordata* (heart-shaped), referring to the nearly heart-shaped sclerite of the male genitalia.

##### Distribution.

Known only from the type locality.

#### Paradiestrammena aquilina

Taxon classificationAnimaliaOrthopteraRhaphidophoridae

Zhu & Tan sp. nov.

A507A789-5C8C-5CF5-A25F-99D83F3FEE8F

https://zoobank.org/8A31BD10-05B9-4792-95AA-BDB96F5B42AA

Figs 2D–F, 3–5

##### Material examined.

***Holotype***: East Malaysia • ♂; Sabah State, Sandakan, Sepilok, Rainforest Discovery Centre; 5.8752°N, 117.9417°E, alt. 20 m, on ground underneath dead log; 19 May 2022; MK Tan & T Robillard leg.; SBH.22.160 (ZRC). ***Paratypes***: East Malaysia • 2♂; Sabah State, Segaliud Lokan Forest Reserve, Leafy Trail; 5.5758°N, 117.6030°E, alt. 80 m, on clayey slope beside trail; 13 July 2026; MK Tan, AA Muhammad & T Robillard leg.; SBH.26.60, 62 (FRC, ZRC).

##### Other material.

East Malaysia • 2♀ nymph and 1♂ nymph; same locality details as paratypes; SBH.26.58, 59, 61 (ZRC) • 2♂ nymph; Sabah State, Segaliud Lokan Forest Reserve; 5.4972°N, 117.5561°E, alt. 255 m, under rock along small creek inside clayey area; 12 July 2026; MK Tan, AA Muhammad & T Robillard leg.; SBH.26.44, 45 (FRC, ZRC).

##### Diagnosis.

The species is similar to *P. gravelyi* from Malay Peninsula in the general habitus and color patterns, but it can be distinguished by the following characters of male genitalia and subgenital plate of female: basal area of sclerite of male genitalia with rather deeper concavity, both sides long (Fig. 2D), while concavity shallower and lateral sides shorter in *P. gravelyi*; and apex of the hooked process of male genitalia rounded (Fig. 2D, F), while apex of the hooked process slightly acute in *P. gravelyi*; the posterior margin of subgenital plate of female is broadly rounded and almost truncated (Fig. 5C), while it has a small process at the posterior margin in *P. gravelyi*.

From *P. sarawakana*, the new species differs by its more intense, larger, dark patches on the pronotum and abdomen; the new species has the anterior and posterior third of the lateral lobes black and the middle third yellow, whereas the lateral lobs are only black along the anterior and posterior margins in *P. sarawakana*.

##### Description.

**Male**. Body medium-sized. Fastigium verticis with two conical tubercles; apices rounded, pointing forwards and downwards. Eyes reniform, protruding forwards. Median ocellus oval, located between antennal sockets. Lateral ocelli nearly circular, situated on lateral surface of basal rostral tubercles. Apical segment of maxillary palp clearly longer than subapical one; apex globular.

**Figure 3.**
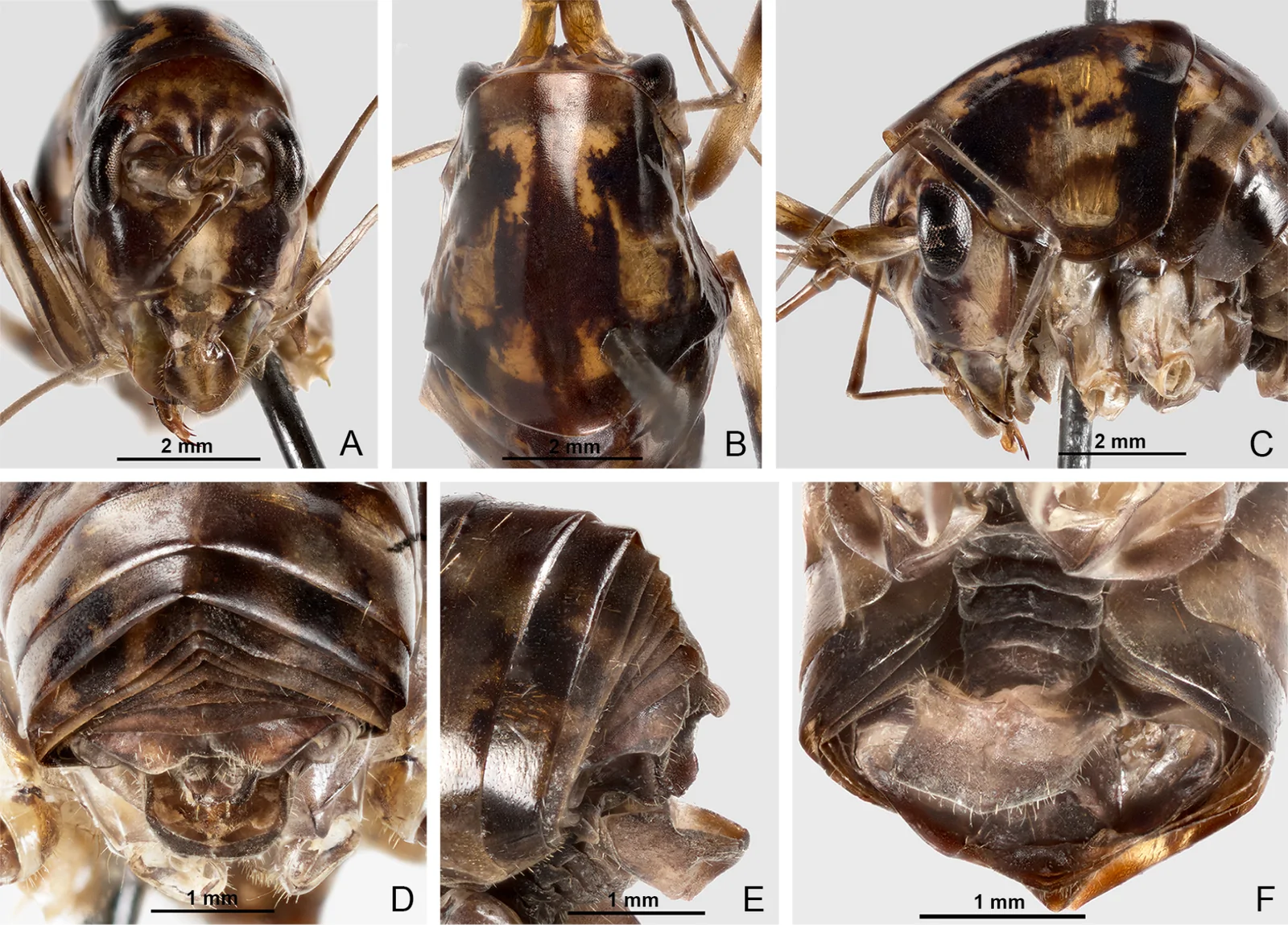
*Paradiestrammena aquilina* Zhu & Tan, sp. nov., ♂ holotype (ZRC). **A–C**. Head and pronotum: **A**. Frontal view; **B**. Dorsal view; **C**. Lateral view. **D–F**. Apex of abdomen: **D**. Dorsal view; **E**. Lateral view; **F**. Ventral view.

Pronotum long; anterior margin of disc straight; posterior margin arcuate; lateral lobe longer than high; ventral margin arched. Mesonotum and metanotum short; posterior margin of mesonotum arcuate; posterior margin of metanotum straight. Fore coxa with one small spine. Fore femur unarmed on ventral surface; internal genicular lobe with one small spine; external genicular lobe with one long spine. Fore tibia with two inner spines and two outer spines on ventral surface, apex with one outer spine on dorsal surface and a pair of spines on ventral surface with one small spine in between. Middle femur unarmed on ventral surface; internal and external genicular lobes with one long spine each. Middle tibia with two inner spines and two outer spines on ventral surface, apex with one dorsal spine and a pair of ventral spines with one small spine in between. Hind femur with four inner spines on ventral surface; internal and external genicular lobes with one small spine each. Hind tibia with 15–16 inner spines and 16–17 outer spines on dorsal surface; subapex with a pair of dorsal spines; apex with a pair of dorsal spines and two pairs of ventral spines, interodorsal spine equal to hind basitarsus. Hind basitarsus with one dorsal spine, with bristles on ventral surface.

**Figure 4.**
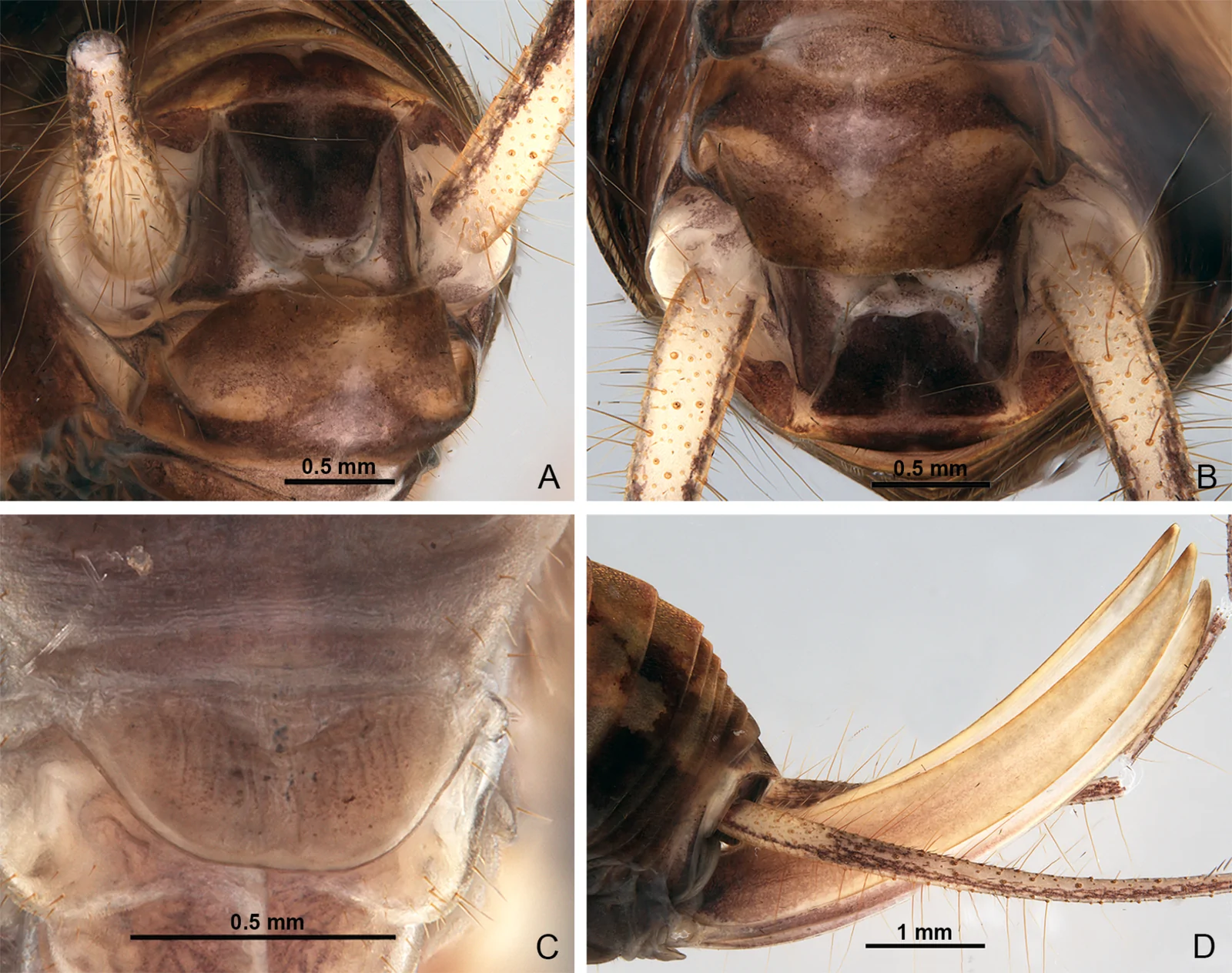
Apical area of abdomen of *Paradiestrammena aquilina* Zhu & Tan, sp. nov., ♂ paratype: **A**. Posterior view; **B**. Ventral view; ♀ nymph: **C**. Ventral view; **D**. Lateral view.

Posterior margin of 10^th^ abdominal tergite slightly concave. Epiproct tongue-shaped. Paraproct triangular in lateral view. Genitalia with sclerite, eagle-wing-shaped, basal area with wide and deep concavity, nearly parallel on both sides, apical area with hooked process, curved upwards, gradually narrowing to apex, apex blunt, pointing to upwards and forwards, with six membranous lobes; dorsomedian lobe broad, apex rounded, dorsolateral lobe cylindrical, nearly equal to dorsomedian lobe, ventromedian lobe shorter than dorsomedian lobe, apex rounded, ventrolateral lobe cylindrical, slightly longer than dorsolateral lobe. Subgenital plate quadrangular, length greater than height, posterior margin straight.

**Figure 5.**
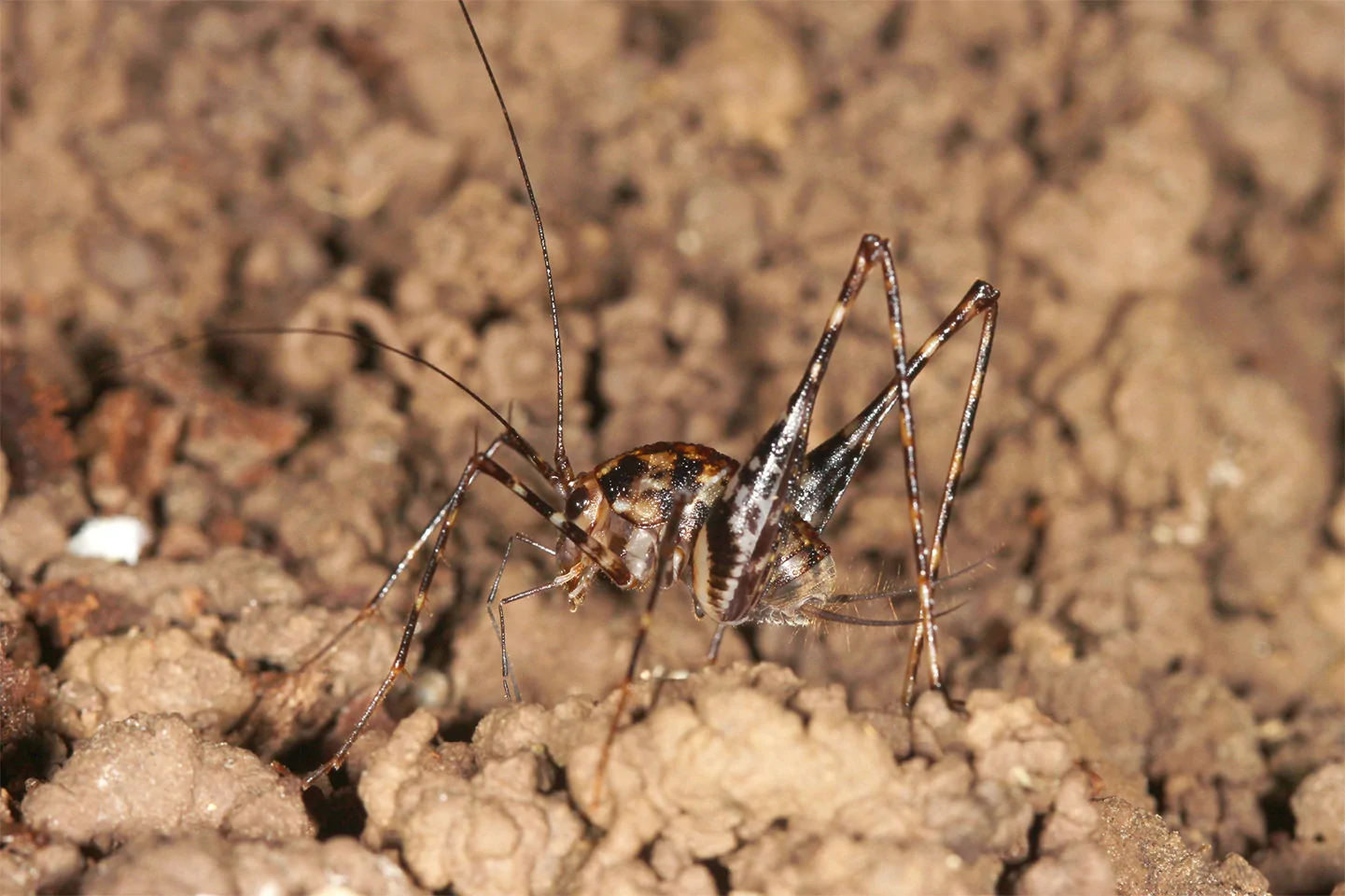
*Paradiestrammena aquilina* Zhu & Tan, sp. nov. ♂ holotype in its natural environment in Sepilok.

**Female**. Nymph with posterior margin of 10^th^ abdominal tergite slightly concave and epiproct tongue-shaped. Subgenital plate transverse, wider than long; posterior margin very broadly rounded and almost truncated. Ovipositor stout, blade-like, with margin smooth, apex subacute.

##### Coloration.

Body brown, with black stripes. Thoracic tergites and first to third abdominal tergites shiny. Lateral margin of fastigium verticis with yellow longitudinal stripes. Eyes black; face with two black longitudinal stripes. Anterior margin of lateral lobe of pronotum with black longitudinal stripe. Legs with black annular pattern, basal half of hind femur with black penniform stripes.

##### Measurements.

Body length: ♂ 8.10–8.85 mm; length of pronotum: ♂ 4.05–4.90 mm; length of fore femur: ♂ 7.25–8.80 mm; length of hind femur: ♂ 15.40–16.85 mm; length of hind tibia: ♂ 16.80–18.10 mm; length of hind basitarsus: ♂ 3.50–4.25 mm.

##### Etymology.

The species name originates from the Latin *aquilin* (eagle-­shaped), in reference to the sclerite of the male genitalia, which is shaped like a giant eagle spreading its wings.

##### Distribution.

Malaysia (Sabah).

##### Remarks.

We observed this species among clayey substrate, often on slopes, in the understory of a sheltered forest. Individuals, both adults and nymph, tend to be found aggregating near each other.

#### Paradiestrammena sabahensis

Taxon classificationAnimaliaOrthopteraRhaphidophoridae

Zhu & Tan sp. nov.

59853103-97B6-57F6-8AF6-FA344A5893FF

https://zoobank.org/410E6896-CFD8-41FC-9EA5-642B33ED5BC2

Figs 2G–I, 6, 7

##### Material examined.

***Holotype***: East Malaysia • ♂; Sabah State, Maliau Basin, Agathis–Nepenthes at 1 km; 4.7027°N, 116.9021°E, alt. 646 m, on base of tree trunk; 14 November 2024, 21h02; MK Tan leg.; SBH.24.88 (FRC). ***Paratype***: East Malaysia • 1♀; Sabah State, same details as holotype; SBH.24.89 (ZRC).

##### Diagnosis.

The species is very similar to *P. aquilina* Zhu & Tan, sp. nov. from Sabah in the general habitus and color patterns, as well as the general shape of the male genitalia (both sclerites and membranous parts), but it can be distinguished from that species by the following characters: distinctly larger size; dorsal process of male genitalia sclerite triangular and at median on the sclerite rather than hooked and on the apical end; sclerite with basal area with wide, shallow concavity and lateral areas stouter; dorsomedian lobe triangular, larger, and more apically protruding. The female also differs in the shape of subgenital plate, which is more acute at the posterior end, and the ovipositor has a crenulated ventral margin at its apex.

**Figure 6.**
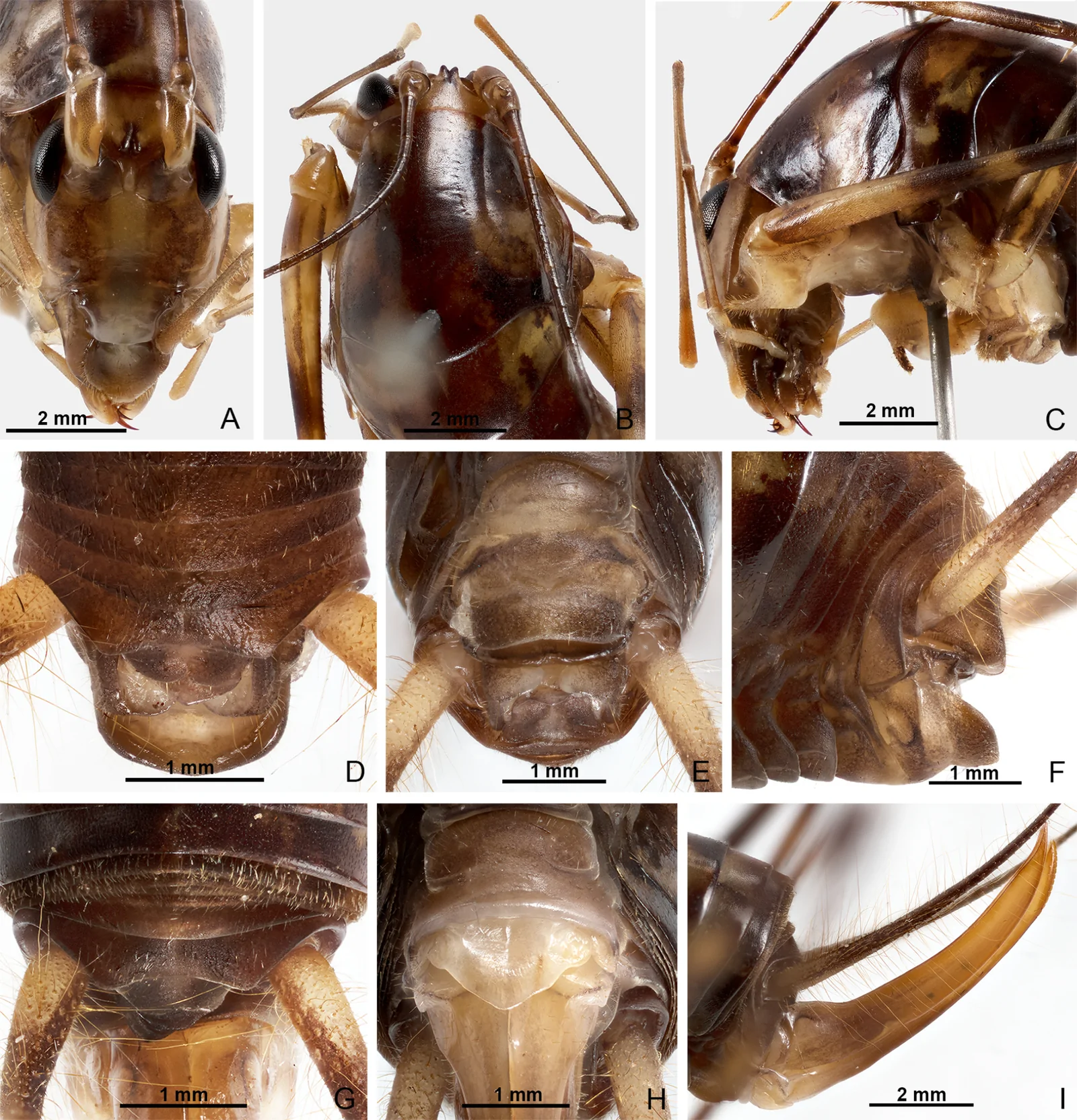
*Paradiestrammena sabahensis* Zhu & Tan, sp. nov. ♂ holotype (FRC). **A–C**. Head and pronotum: **A**. Frontal view; **B**. Dorsal view; **C**. Lateral view. **D–F**. Apex of abdomen: **D**. Dorsal view; **E**. Ventral view; **F**. Lateral view. ♀ paratype (ZRC). **G–I**. Apex of abdomen: **G**. Dorsal view; **H**. Ventral view; **I**. Lateral view.

From *P. gravelyi*, the new species differs by a larger habitus. It also differs in the sclerite of the male genitalia and subgenital plate of the female: process on apical area of sclerite triangular (Fig. 2I), but hooked in *P. gravelyi*; subgenital plate triangular (Fig. 6H), but quadrangular and with a small process at posterior margin in *P. gravelyi*.

##### Description.

**Male**. Body large for the genus. Fastigium verticis with two conical tubercles; apices rounded, pointing forwards and downwards. Eyes reniform, protruding forwards. Median ocellus oval, between antennal sockets. Lateral ocelli nearly circular, on lateral surface of basal rostral tubercles. Apical segment of maxillary palp obviously longer than subapical one; apex globular.

**Figure 7.**
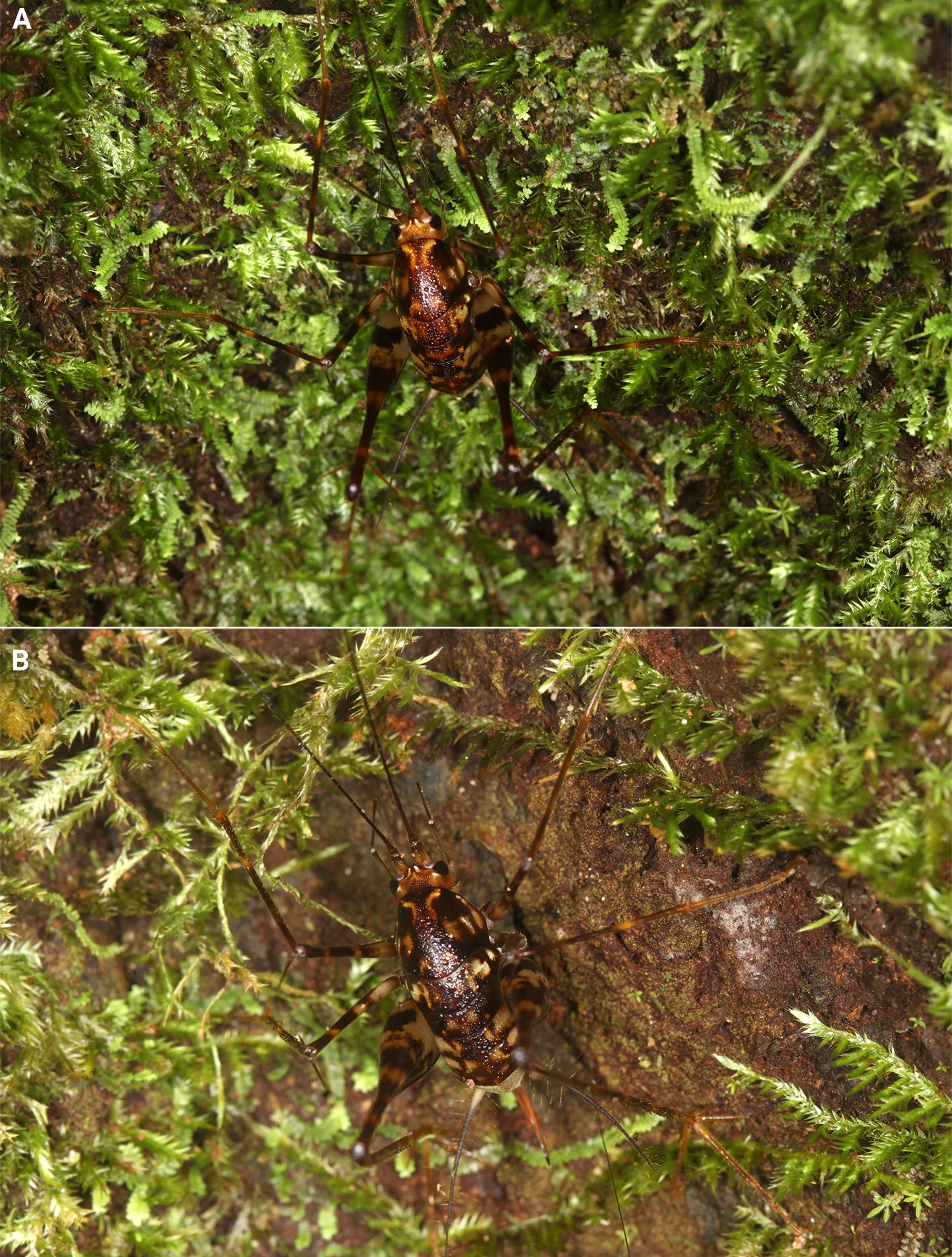
*Paradiestrammena sabahensis* Zhu & Tan, sp. nov. in its natural environment in Sabah. **A**. Male; **B**. Female.

Pronotum long; anterior margin of disc straight; posterior margin arcuate; lateral lobe longer than high; ventral margin arched. Mesonotum and metanotum short; posterior margin of mesonotum arcuate; posterior margin of metanotum straight. Fore coxa with one small spine, setose and apex blunt. Fore femur unarmed on ventral surface; internal genicular lobe with one small spine; external genicular lobe with one long spine. Fore tibia with two inner spines and two outer spines on ventral surface; apex with one outer spine on dorsal surface and a pair of spines on ventral surface with one small spine in between. Middle femur unarmed on ventral surface; internal and external genicular lobes with one long spine each. Middle tibia with two inner spines and two outer spines on ventral surface; apex with one dorsal spine and a pair of ventral spines with one small spine in between. Hind femur with two to four inner spines on ventral surface; internal and external genicular lobes with one small spine each. Hind tibia with 15–16 inner spines and 16–17 outer spines on dorsal surface; subapex with a pair of dorsal spines; apex with a pair of dorsal spines and two pairs of ventral spines; interodorsal spine equal to hind basitarsus. Hind basitarsus with one dorsal spine, with bristles on ventral surface.

Posterior margin of 10^th^ abdominal tergite faintly concave. Epiproct tongue-shaped, emarginated at the apex. Paraproct triangular in lateral view and rectangular in dorsal view. Genitalia with sclerite, eagle-wing-shaped, basal area with wide and shallow concavity, stout and nearly parallel on both sides, medial area with triangular process, curved upwards, apex acute, pointing to upwards; with six membranous lobes: dorsomedian lobe large and triangular with apical area rounded; dorsolateral lobe finger-like, distinctly shorter than dorsomedian lobe, at middle slightly bent and apex rounded; ventromedian lobe shorter than dorsomedian lobe, apex narrowly emarginated; ventrolateral lobe cylindrical, slightly shorter than dorsolateral lobe with apex truncated and more setose. Subgenital plate quadrangular, length greater than height, posterior margin slightly convex.

**Female**. Similar to male in habitus. Posterior margin of 10^th^ abdominal tergite slightly concave and epiproct tongue-shaped. Subgenital plate triangular, wider than long; basally with small but broad lateral lobe; posterior end subacute. Ovipositor stout, blade-like, dorsal valves very slender at apex, longer than ventral valves; valves with margins smooth, except ventral margin of ventral valves crenulated; apices of dorsal valves subacute, of ventral valves acute.

##### Coloration.

Very similarly colored as *P. aquilina* Zhu & Tan, sp. nov.

##### Measurements.

Body length: ♂ 11.8 mm, ♀ 10.8 mm; length of pronotum: ♂ 4.6 mm, ♀ 4.4 mm; length of fore femur: ♂ 11.1 mm, ♀ 10.6 mm; length of hind femur: ♂ 19.8 mm, ♀ 20.0 mm; length of hind tibia: ♂ 21.3 mm, ♀ 22.7 mm; length of hind basitarsus: ♂ 4.9 mm, ♀ 4.9 mm; ovipositor length 7.1 mm.

##### Etymology.

The specific name originates from the type locality.

##### Distribution.

Known only from the type locality.

### Key to species of *Paradiestrammena*, based on male characters*

**Table:** 

1	Apical area of dorso-median lobe of male genitalia concave (Fig. 2A)	**2**
–	Apical area of dorso-median lobe of male genitalia rounded	**5**
2	Basal area of sclerite of male genitalia concave (Fig. 2A)	**3**
–	Basal area of sclerite of male genitalia nearly straight	**4**
3	Sclerite of male genitalia distinctly larger and wider, subapical area with longer hooked process (Fig. 2A, C)	***P. cordata* sp. nov**.
–	Sclerite of male genitalia smaller and narrower, apical area slightly swollen and curved upwards	***P. autumnalis***
4	Basal area of sclerite of male genitalia narrow, nearly half of the high, apical area slightly curved upwards	***P. contumi***
–	Basal area of sclerite of male genitalia wide, slightly shorter than high, apical area slightly curved downwards	***P. maculata***
5	Apical area of sclerite of male genitalia with long process, hooked or spine-shaped	**6**
–	Apical area of sclerite of male genitalia slightly curved upwards, apices rounded	**9**
6	Basal area of sclerite of male genitalia with a small concavity, apical area with long, thin, spine-shaped process	***P. tarbinskyi***
–	Basal area of sclerite of male genitalia with a wide and deep concavity, apical area with hooked or triangular process (Fig. 2D, F, G, I)	**7**
7	Basal area of sclerite of male genitalia with rather longer lateral clade, and apex of process rounded (Fig. 2F)	***P. aquilina* sp. nov**.
–	Basal area of sclerite of male genitalia with shorter lateral clade, and apex of process acute (Fig. 2I)	**8**
8	Apical area of sclerite of male genitalia with hooked process	***P. gravelyi***
–	Apical area of sclerite of male genitalia with triangular process	***P. sabahensis* sp. nov**.
9	Apical area of sclerite of male genitalia slightly straight	***P. vitalisi***
–	Apical area of sclerite of male genitalia rounded	**10**
10	Sclerite of male genitalia nearly triangular, lateral margin slightly straight	***P. trigona***
–	Lateral margin of sclerite of male genitalia protuberant or concave	**11**
11	Lateral margin of sclerite of male genitalia concave	***P. xima***
–	Sclerite of male genitalia nearly heart-shaped, lateral margin protuberant	***P. vernalis***

* *Paradiestrammena enata*, *P. sarawakana*, *P. storozhenkoi* are only known as female, and the male of *P. mistshenkoi* is nymph. Therefore, these four species are not included in this key.

## Discussion

The genus *Paradiestrammena* comprises 16 species, distributed mainly in Vietnam, Cambodia, Malaysia, Thailand, Laos, and China (Cigliano et al. 2026; present work). It closely resembles *Atachycines* and *Eutachycines*, which belong to the same tribe, in terms of body size and morphology of the male genitalia. All three genera are predominantly distributed in Southeast Asia, a pattern of distribution that markedly distinguishes them from related genera. The taxonomic placement of several species among these three genera has undergone repeated revision (Karny 1920, 1925; Chopard 1921; Storozhenko 1990; Sugimoto and Ichikawa 2003), but the generic placement of species in these three genera and the phylogenetic relationships among them is still unclear. The taxonomy of these three genera has remained problematic for nearly two decades. Here, we examined cave cricket specimens deposited in the Lee Kong Chian Natural History Museum, National University of Singapore, and identified three new species of the genus *Paradiestrammena* collected from Borneo, Malaysia. Further investigation is required to clarify the phylogenetic relationships among these genera and to reassess the validity of their current species-level classifications.

## Supplementary Material

XML Treatment for Paradiestrammena

XML Treatment for Paradiestrammena cordata

XML Treatment for Paradiestrammena aquilina

XML Treatment for Paradiestrammena sabahensis

## Additional information

### Artificial Intelligence (AI) use

The authors accept full responsibility for the content of the manuscript, including the disclosure of any use of AI.

No AI tools were used in the preparation of this manuscript.

### Author ORCIDs

Q. Zhu https://orcid.org/0000-0001-8187-1286

A.A. Muhammad https://orcid.org/0000-0001-5772-1149

T. Robillard https://orcid.org/0000-0002-2177-9549

D.F.B.A. Damit https://orcid.org/0009-0008-2925-7623

R. Japir https://orcid.org/0009-0004-1879-6550

A.Y.C. Chung https://orcid.org/0000-0002-9529-4114

F. Shi https://orcid.org/0000-0002-4885-9012

M.K. Tan https://orcid.org/0000-0002-4324-6305

### Data availability

All of the data that support the findings of this study are available in the main text.
